# Humanitarian Love in Values-Based Practice and Health Professionals’ Psychosocial Outcomes: Systematic Review

**DOI:** 10.1192/j.eurpsy.2024.574

**Published:** 2024-08-27

**Authors:** A. L. Batiridou, E. Dragioti, S. Mantzoukas, M. Gouva

**Affiliations:** ^1^Research Laboratory of Psychology of Patients, Families and Health Professionals, Department of Nursing, School of Health Sciences; ^2^Research Laboratory of Integrated Health, Care and Well-being, Department of Nursing, School of Health Sciences, University of Ioannina, Ioannina, Greece

## Abstract

**Introduction:**

The literature on Values-Based Practice often neglects the significance of love in therapeutic interactions, sometimes treating it as taboo or crossing professional boundaries.

**Objectives:**

This systematic review investigates the role of humanitarian love in the lives of healthcare professionals and its psychosocial impact, aiming to establish it as a core value in values-based practice.

**Methods:**

We conducted a PRISMA 2020-compliant systematic review, searching databases (CINAHL, PubMed, Scopus) from inception to April 3, 2023, using PEO elements: health professionals (P), love (E), psychosocial impact (O). Two independent reviewers conducted screening, data extraction, and bias assessment. A narrative synthesis of the data was applied. The selection process is presented in Figure 1.

**Results:**

Eight articles met the inclusion criteria, comprising 1,948 participants (median age: 28.55). Humanitarian love encompassed compassionate love, self-compassion, and affection. Humanitarian love showed a negative correlation with burnout, compassion fatigue, self-judgment, and secure attachment, while positively correlating with professional well-being, professional commitment, self-care, patience, diversity acceptance, spirituality, self-kindness, and ethical values. Humanitarian love significantly influenced healthcare professionals’ psychosocial well-being. The main outcomes are presented in Figure 2.

**Image:**

**Figure:**
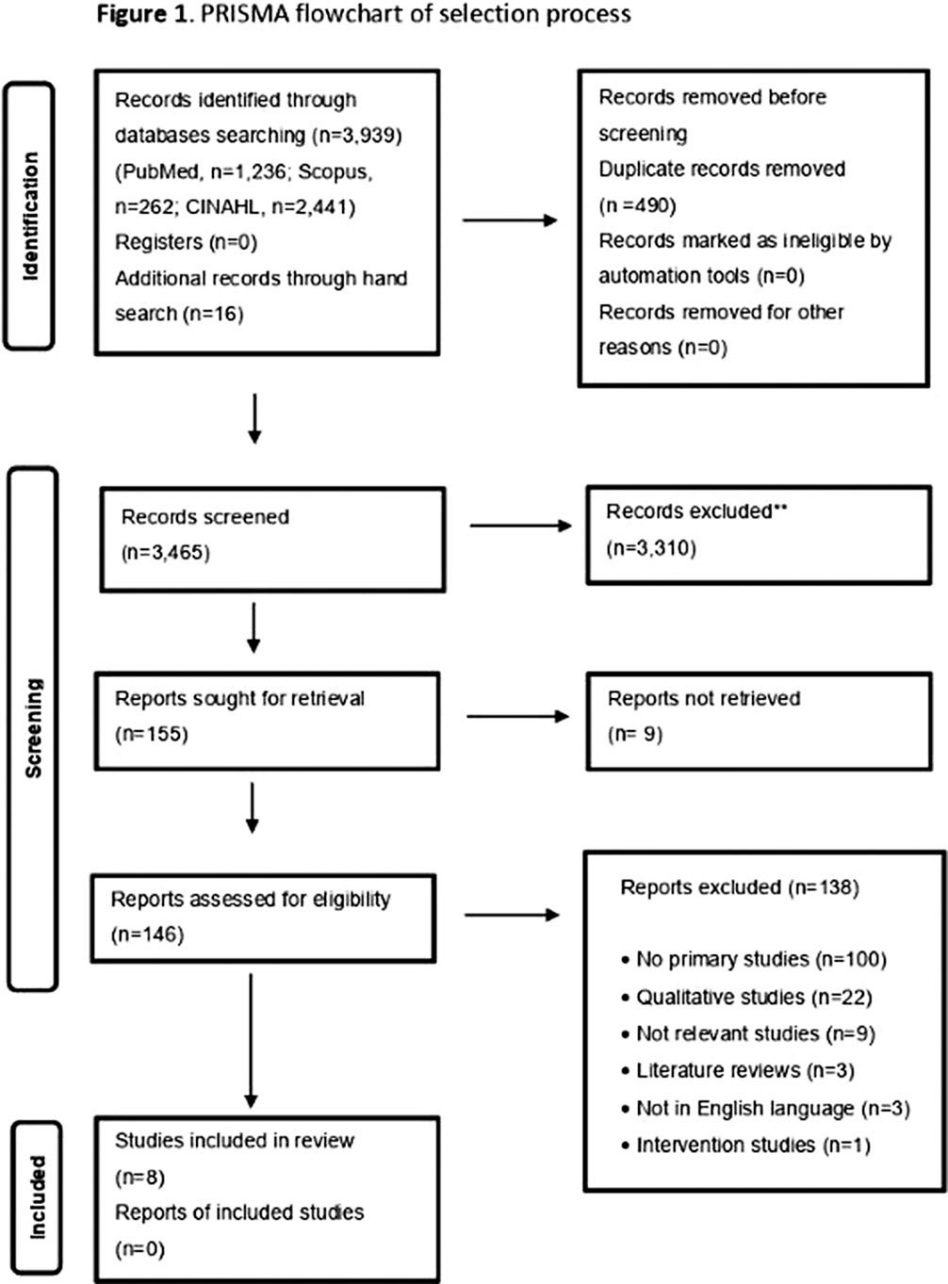

**Image 2:**

**Figure:**
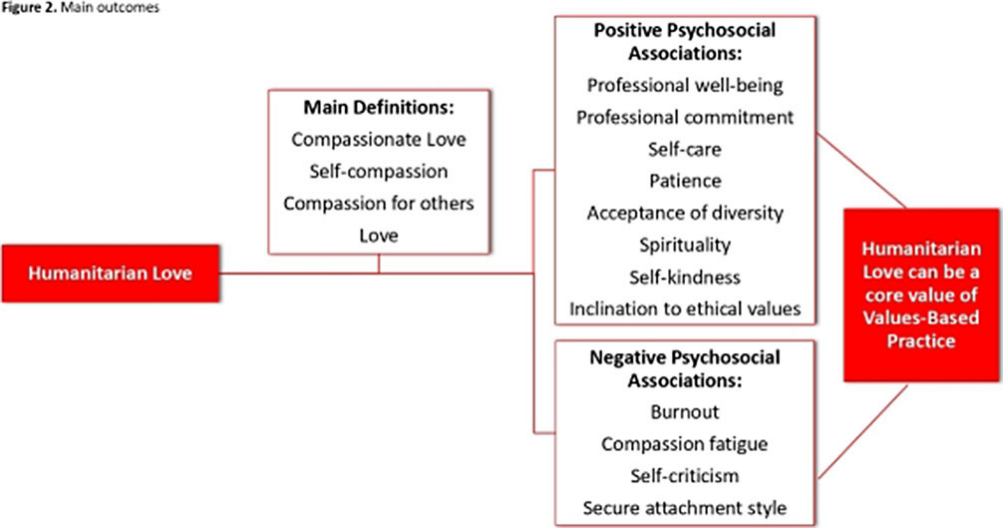

**Conclusions:**

This review highlights humanitarian love’s potential to enhance the psychosocial well-being of healthcare professionals and emphasizes its significance as a core value in values-based practice. Cultivating humanitarian love among healthcare professionals through research and interventions could bolster their resilience, job satisfaction, and overall fulfillment in their roles.

**Disclosure of Interest:**

None Declared

